# Androsterone glucuronide to dehydroepiandrosterone sulphate ratio is discriminatory for obese Caucasian women with polycystic ovary syndrome

**DOI:** 10.1186/s12902-017-0177-3

**Published:** 2017-05-19

**Authors:** Li-Wei Cho, Thozhukat Sathyapalan, Eric S Kilpatrick, Brian G Keevil, Adrian G Miller, Anne M Coady, Lina Ahmed, Stephen L Atkin

**Affiliations:** 1 Department of Endocrinology, Changi General Hospital, ᅟSingapore, Singapore; 2 Department of Academic Diabetes, Endocrinology and Metabolism, University of Hull, ᅟHull, UK; 3 Sidra Medical Research Centre, PO Box 24699, ᅟDoha, Qatar; 4 Department of Clinical Biochemistry, Wythenshawe Hospital, Manchester, UK; 5 Department of Radiology, Hull Royal Infirmary, ᅟHull, UK; 6 Weill Cornell Medicine Qatar, PO Box 24144, Doha, Qatar

**Keywords:** Polycystic ovary syndrome, Androsterone glucuronide, DHEAS

## Abstract

**Background:**

Androsterone glucuronide (ADTG) concentrations have been suggested as a marker of the effects of androgens at the target tissue level. As the mechanism for hyperandrogenemia in obese and nonobese polycystic ovary syndrome (PCOS) may differ, this study compared the different androgen parameters in non-obese compared to obese women with PCOS, and in normal subjects.

**Methods:**

Eleven non-obese and 14 obese women with PCOS were recruited and compared to 11 control women without PCOS. Total testosterone, dehydroepiandrosterone sulphate (DHEAS), ADTG, and androstenedione were analysed using gold standard tandem mass spectrometry, and the free androgen index (FAI) was calculated.

**Results:**

Total testosterone, ADTG and androstendione levels did not differ between non-obese (body mass index (BMI) ≤25 kg/m^2^) and obese PCOS (BMI >25 kg/m^2^) but all were significantly higher than for controls (*p* < 0.01). The ADTG to DHEAS ratio was significantly elevated 39 ± 6 (*p* < 0.01) in obese PCOS in comparison to non-obese PCOS and controls (28 ± 5 and 29 ± 4, respectively). The free androgen index (FAI) and insulin resistance (HOMA-IR) were significantly higher in obese PCOS compared to non-obese PCOS and controls (*p* < 0.01). DHEAS was significantly higher in the non-obese versus obese PCOS (*p* < 0.01). All androgen parameters were significantly lower and sex hormone binding globulin (SHBG) significantly higher in normal subjects compared to those with obese and non-obese PCOS.

**Conclusions:**

The ADTG:DHEAS ratio was significantly elevated in obese PCOS compared to non-obese PCOS and controls suggesting that this may be a novel biomarker discriminatory for obese PCOS subjects, perhaps being driven by higher hepatic 5α reductase activity increasing ADTG formation in these women.

## Background

Polycystic ovary syndrome is one of the most common endocrine disorders and affects 7–9% of reproductive-aged women [1–3]. Hyperandrogenism is central to the diagnosis of PCOS in the NIH, the Rotterdam consensus and the Androgen excess society [4] criteria, but agreement is still lacking on the best androgen measurement in women with PCOS who suffer from a high incidence of acne and hirsutism, reflecting increased androgen secretion.

With the advent of liquid chromatography tandem mass spectroscopy the accurate measurement of low serum levels of androgens, their precursors and their metabolites is now possible, allowing the role of androgens in PCOS to be studied with sufficient sensitivity and specificity [5]. Recently it was proposed that the testosterone to dihydrotestosterone ratio may be used as a biomarker particularly for those with an adverse metabolic phenotype [6]. Androsterone glucuronide (ADTG) reflects adrenal androgen secretion from hepatic 5α-reductase activity, and to a lesser extent peripheral 5α-reductase activity, that converts dehydroepiandrosterone sulphate (DHEAS) to ADTG [7, 8]. Thus, DHEAS and to a lesser amount DHEA (approximately 20%) are converted to ADTG by hepatic and peripheral 5α-reductase and therefore the concentration of ADTG will reflect both DHEAS levels and 5α-reductase activity. ADTG has been reported to be a more reliable marker for the effects of androgen at the target tissue level and studies have shown that ADTG is significantly elevated in hirsute compared to non-hirsute women with PCOS [9–11]. Previously ADTG has been measured by immunoassay that may be inaccurate due to cross-reactivity with other androgen metabolites such as DHEAS [12, 13], circumvented by tandem mass spectrometry.

Some studies have reported increased hyperandrogenaemia in obese compared with non-obese women with PCOS [14] suggesting that the mechanism for development of hyperandrogenism may differ in these two groups of subject. The aim of this study was to compare different androgen parameters of testosterone, DHEAS, ADTG and androstenedione in nonobese compared to obese women with PCOS, and compared to control subjects.

## Methods

Eleven nonobese [BMI 22.9 ± 1.4 kg/m^2^] and 14 age-matched obese [BMI 36.8 ± 4.8 kg/m^2^] Caucasian women with a diagnosis of PCOS based on all three diagnostic criteria of the Rotterdam consensus, namely clinical (Ferriman-Gallwey score > 8) and biochemical evidence of hyperandrogenaemia, oligomenorrhea or amenorrhoea (cycle length, <21 day or >35 days; <8 cycles per year) and polycystic ovaries on transvaginal ultrasound. Non classical 21-hydroxylase deficiency, hyperprolactinemia, and androgen secreting tumours were excluded by appropriate tests before the diagnosis of PCOS was made. Eleven Caucasian women with normal menstrual cycle and no evidence of clinical or biochemical hyperandrogenism were recruited as controls by advert. All subjects were recruited in Hull. Diabetes was excluded in all subjects by a 75 g oral glucose tolerance test. No subject was on any medication that could alter insulin resistance at the time of the study or within the preceding three months of entering the trial. There was no evidence of non-alcoholic fatty liver disease on abdominal ultrasound examination. Subjects with PCOS were recruited from the endocrine clinic at Hull Royal Infirmary, UK. This was a prospective study that enrolled all patients that fulfilled the inclusion and exclusion criteria and as a consequence the 2 groups were not age matched. All subjects gave their informed consent and the study was approved by the Hull and East riding ethics committee.

Fasting venous blood was collected into serum gel tubes (Becton Dickinson, Cowley, Oxfordshire, UK) at the same time each day (0800–0900). Samples were separated by centrifugation at 2000 g for 15 min at 4 °C, and stored at –20 °C within 1 h of collection.

### Laboratory analysis

Before analysis, all the serum samples were thawed and thoroughly mixed. Serum insulin was assayed using a competitive chemiluminescent immunoassay performed on a Siemens Immulite 2000 analyser (Siemens Ltd, Frimley, UK), using the manufacturer’s recommended protocol. There was no stated cross-reactivity with proinsulin. Plasma glucose was measured using a Synchron LX 20 analyser (Beckman-Coulter, High Wycombe, UK), using the manufacturer’s recommended protocol. The coefficient of variation for this assay was 1.2% at a mean glucose value of 5.3 mmol/l (94.6 mg/dl). The IR was calculated using the homeostasis model assessment (HOMA) method (HOMA-IR = (insulin μIU/mL x glucose mmol/l)/22.5) [14]. Serum testosterone [15], androstenedione [16], DHEAS [7, 8], and ADTG [17] were all measured by isotope dilution liquid chromatography- tandem mass spectrometry (Waters Corporation, Manchester, UK) using fully validated methods (17). SHBG was measured by chemiluminescent detection on the Siemens Immulite 2000 analyzer using the manufacturer’s recommended protocol. The free androgen index was obtained as the quotient 100 testosterone/SHBG.

### Statistical analysis

Statistical analysis was performed using SPSS for Windows, version 22.0. The data was normally distribution between individuals and none violated the assumptions of normality when tested using the Kolmogorov-Smirnov test and therefore are expressed as mean ± SD. One way ANOVA was undertaken with post hoc testing between groups. For all analysis, a two-tailed *P* < 0.05 was considered to indicate statistical significance.

## Results

Baseline parameters of subjects are shown in Table 1. Obese and non-obese PCOS groups were age matched and were significantly younger than the control subjects. Weight did not differ significantly between the nonobese and control subjects but both differed significantly from the obese PCOS group.

**Table 1:** Baseline parameters of non-obese and obese patients with PCOS and normal controls (mean ± SD), showing the elevated androgen levels found in PCOS subjects, the elevated ADTG: DHEAS ratio and increased insulin resistance found for the obese PCOS patients

Parameters	Nonobese (*n*=11)	Obese (*n*=14)	Controls (*n*=11)	*p* value between nonobese and obese PCOS	*p* value between obese PCOS and controls	*p* value between nonobese PCOS and controls
Age (years)	23.3 ± 4.5	26.0 ± 4.1	32.6 ± 4.9	0.13	<0.01	0.01
BMI (kg/m^2^)	22.9 ± 1.4	36.8 ± 4.8	26.0 ± 4.7	<0.01	<0.01	0.051
Total Testosterone (nmol/L)	1.7 ± 0.7	1.6 ± 0.7	1.0 ± 0.4	0.73	<0.02	<0.02
SHBG (nmol/L)	42.5 ± 16.3	25.4 ± 10.6	61.4 ± 21.8	<0.01	<0.01	<0.03
FAI	4.3 ± 1.7	7.3 ± 4.4	1.9 ± 1.0	<0.03	<0.01	<0.01
DHEAS (μmol/L)	7.6 ± 2.3	5.5 ± 1.7	3.3 ±1.4	<0.05	<0.01	<0.01
ADTG (nmol/L)	213.7 ± 82.3	216.9 ± 121.7	97.7 ± 52.0	0.9	<0.01	<0.01
ADTG/DHEAS ratio (nmol/umol)	28 ± 5	39 ± 6	29 ± 4	<0.01	<0.01	0.85
Androstenedione (nmol/L)	7.3 ± 2.8	6.9 ± 3.6	3.2 ± 1.5	0.73	<0.01	<0.01
HOMA-IR	1.6 ± 0.7	4.4 ± 2.8	1.6 ± 0.7	<0.01	<0.01	1.00

*SHBG* sex hormone binding globulin, *FAI* free androgen index, *DHEAS* dehydroepiandrosterone sulphate, *ADTG* androsterone glucuronide, *HOMA-IR* homeostasis model assessment method for insulin resistance

ADTG and androstendione levels did not differ between non-obese (BMI ≤25 kg/m^2^) and obese PCOS (BMI >25 kg/m^2^) but both were significantly higher than for controls (*p* < 0.01). However, the ADTG to DHEAS ratio was significantly elevated 39 ± 6 (*p* < 0.01) in obese PCOS in comparison to nonobese PCOS and controls (28 ± 5 nmol/umol and 29 ± 4 nmol/umol, respectively). DHEAS was significantly higher in the nonobese versus obese PCOS and controls (*p* < 0.01). Both non-obese and obese PCOS were equally hyperandrogenic as measured by total testosterone (*p* = 0.73), but the FAI (*p* < 0.01) and insulin resistance (HOMA-IR) (*p* < 0.01) was significantly higher in obese PCOS. HOMA-IR did not differ between non-obese PCOS and control subjects. All androgen parameters were significantly lower and SHBG significantly higher in normal subjects (*p* < 0.01) compared to those with obese and nonobese PCOS.

## Discussion

This study showed that using state of the art measurement that the ADTG:DHEAS ratio was significantly elevated in obese PCOS compared to non-obese PCOS and to controls subjects, whilst the latter two groups did not differ, suggesting that this may be a novel biomarker and discriminatory for PCOS in obesity. Thus, in the case of diagnostic uncertainty, the presence of both obesity a raised ADTG:DHEAS ratio could help confirm the diagnosis of PCOS, and the ratio could be an indirect measure of 5α-reductase activity that may identify those individuals whose insulin resistance is having a marked effect on their androgen metabolism. Total testosterone, ADTG and androstenedione levels did not differ between obese and non obese PCOS patients, though all were significantly higher than that of the control subjects. DHEAS levels were higher in the non-obese PCOS subjects than obese PCOS and control subjects, data are in accord with that shown by Silfen and colleagues [6]. The raised DHEAS levels should have reflected in a higher ADTG levels that derive from DHEAS but this was not seen and indeed the conversion ratio from DHEAS was the same as the normal controls. It has been shown that conversion of DHEAS to ADTG occurs mainly through hepatic 5α-reductase activity, and to a lesser extent peripheral 5α-reductase activity [9–11]. It is reported that hepatic 5α-reductase activity is increased in insulin resistant states [18] and it can be seen from the results that the obese PCOS patients were significantly more insulin resistant than the non-obese PCOS patients and the normal controls (the HOMA-IR did not differ between the non-obese PCOS and normal subjects) suggesting that the increased insulin resistance in obesity may be driving the hepatic 5α-reductase activity converting DHEAS to ADTG. The enhanced insulin resistance in obesity was reflected in the decrease in the SHBG levels particularly in the obese PCOS subjects resulting in an increased FAI. There is little data on the role ADTG in PCOS and androgen metabolism, however this study showing the increased ADTG: DHEAS ratio in obese PCOS using state of the art measurement free from assay interference addresses the discrepancy noted in one study that it was not correlated with PCOS [8, 18], and with another suggesting that ADTG levels are increased [13].

It was recently proposed that the testosterone to dihydrotestosterone ratio may be used as a biomarker particularly for those with an adverse metabolic phenotype [18]. It would be of value to prospectively determine if the ADTG: DHEAS ratio would add additional value as an additional biomarker for the metabolic phenotype.

PCOS is a condition that encompasses a spectrum of symptoms and presentations that may vary from one patient to another. It is still unknown whether non-obese and obese PCOS share the same pathology or in fact there may be additional factors, whether external or internal, that triggers the progression from one condition to the other. ADTG is significantly elevated in hirsute compared to non-hirsute women with PCOS and it has been suggested that ADTG is thought to be a more reliable parameter of the effects of androgen at the target tissue level [19].

Androgen levels were significantly higher in PCOS compared to controls for total testosterone, FAI, DHEAS, ADTG and androstenedione in accord with the studies showing the androgenic burden in PCOS [20]. It is reported that androgen levels fall with age towards the menopause though still remain elevated after the menopause. In this study the control patients age were higher than the obese and non-obese PCOS patients; however, the estimated decrease in androgens with that age difference would have been minimal and insufficient to account for the differences seen.

There is concern that hyperandrogenism is associated with a more atherogenic phenotype leading to increase future atherosclerosis in women. Recent studies have shown that whilst there appears to be a relationship of total testosterone to intima media thickness and androstenedione and DHEAS to cardiac changes, longitudinal analysis showed no association of total testosterone to subclinical cardiovascular disease over a 5 year period.

The strengths of this study include well-defined population of subjects and measurement of androgens using gold standard tandem mass spectrometry. Despite the very significant androgen changes, nonetheless the small number of subjects limited this study and there is a need to repeat the measures using stratified weight and age match cohorts particularly weight matching the control subjects for the obese and non obese PCOS groups. Additional measurements of DHEA (the second largest source of ADTG) in blood and for the androstenedione:etiocholanolone ratio in urine as a marker of 5 alpha reductase would be useful to evaluate.

## Conclusions

The ADTG: DHEAS ratio was significantly elevated in obese PCOS compared to non-obese PCOS and control subjects suggesting that this may be a novel biomarker to identify obese PCOS patients, with ADTG formation perhaps being driven by higher hepatic 5α reductase activity reflecting the increased insulin resistance seen in the obese PCOS group.

## Abbreviations

ADTG: Androsterone glucuronide
BMI: Body mass index
DHEAS: Dehydroepiandrosterone sulphate
FAI: Free androgen index
HOMA: Homeostasis model assessment
PCOS: Polycystic ovary syndrome
SHBG: Sex hormone binding globulin
